# Neurodevelopmental outcomes of extremely preterm infants fed an exclusive human milk-based diet versus a mixed human milk + bovine milk-based diet: a multi-center study

**DOI:** 10.1038/s41372-022-01513-3

**Published:** 2022-09-28

**Authors:** Amy B. Hair, Aloka L. Patel, Ursula Kiechl-Kohlendorfer, Jae H. Kim, Richard J. Schanler, Keli M. Hawthorne, Elena Itriago, Steven A. Abrams, Cynthia L. Blanco

**Affiliations:** 1 Department of Pediatrics, Division of Neonatology, Baylor College of Medicine, Texas Children’s Hospital, Houston, TX USA; 2 Department of Pediatrics, Neonatology, Rush University Medical Center, Chicago, IL USA; 3 Department of Pediatrics II (Neonatology), Medical University of Innsbruck, Innsbruck, Austria; 4 Department of Pediatrics, Perinatal Institute, Division of Neonatology, Cincinnati Children’s Hospital Medical Center, University of Cincinnati College of Medicine, Cincinnati, OH USA; 5 Department of Pediatrics, Neonatal Perinatal Medicine, Zucker School of Medicine at Hofstra/Northwell, New Hyde Park, NY USA; 6 Department of Pediatrics, Dell Medical School, University of Texas at Austin, Austin, TX USA; 7 Department of Pediatrics, University of Texas Health Science Center, San Antonio, TX USA

**Keywords:** Intestinal diseases, Nutrition disorders

## Abstract

**Objective:**

The objective of this multi-center study was to compare, in infants ≤1250 g birth weight (BW) with neurodevelopmental assessment at 18–22 months of corrected age (CA), whether their neurodevelopmental outcomes differed based on exposure to an exclusive human milk-based (HUM) or to a bovine milk-based fortifier and/or preterm formula (BOV).

**Study Design:**

Retrospective multi-center cohort study of infants undergoing neurodevelopmental assessment as to whether HUM or BOV exposure related to differences in outcomes of infants at 18–22 months CA, using the Bayley Scales of Infant Development III (BSID-III). BSID-III cognitive, language, and motor scores were adjusted for BW, sex, study site, and necrotizing enterocolitis.

**Results:**

252 infants from 6 centers were included. BSID-III cognitive scores were higher in the HUM group (96.5 ± 15.1 vs 89.6 ± 14.1, adjusted *p* = 0.0001). Mean BSID-III language scores were 85.5 ± 15.0 in HUM and 82.2 ± 14.1 in BOV (adjusted *p* = 0.09). Mean BSID-III motor scores were 92.9 ± 11.7 in HUM and 91.4 ± 14.6 in BOV (adjusted *p* = 0.32).

**Conclusion:**

In this cohort of infants undergoing neurodevelopmental assessment, infants receiving HUM diet had significantly higher cognitive BSID-III scores at 18–22 months CA. Further investigation is needed of this potential for HUM to positively influence infant cognitive outcomes.

## Introduction

An exclusive human milk-based diet, comprised of mother’s own milk, supplemented with donor human milk when needed, fortified with a pasteurized donor human milk-derived fortifier (HUM), has been shown to reduce the incidence of serious medical conditions in preterm infants ≤1250 grams (g) birth weight (BW) [1–3]. Reports of beneficial inpatient outcomes include decreased rates of necrotizing enterocolitis (NEC), decreased parenteral nutrition days, and decreased mortality when compared to a mixed bovine diet including mother’s milk and bovine milk fortifier and/or formula (BOV) in extremely preterm infants [1–3].

While there are studies on the short-term outcomes of infants who received HUM compared to BOV diet in the neonatal intensive care unit (NICU), there are limited post-discharge neurodevelopmental outcome data for infants who were fed a HUM diet [4–6]. Multiple studies have shown the positive dose-dependent relationship between percent intake of MOM and neurodevelopmental outcomes in preterm infants in infancy and beyond [7–12]. While MOM has shown a positive benefit, studies have shown that there is no significant difference in neurodevelopmental outcomes of preterm infants receiving donor human milk compared to preterm infant formula [13]. In a double blinded, randomized controlled trial, O’Connor et al. showed that preterm infants who received supplemental (when MOM’s milk was unavailable) donor human milk compared to formula did not have an improvement in neurodevelopmental outcomes at 18 months CA [14]. Of note, preterm infants in this study received a high provision of MOM with limited supplemental donor human milk or formula. There are no studies evaluating outcomes of infants receiving a majority of their diet from donor human milk. Current studies that have compared HUM to BOV diet have shown no differences in neurodevelopmental outcomes at 18 months CA; however fortification strategies used in these studies [4, 5] do not reflect early fortification and rapid advancement of caloric content of feeds using a pasteurized donor human milk-based fortifier (HUM).

The objective of this multi-center study was to compare, in infants ≤1250 g BW with neurodevelopmental assessment at 18–22 months CA, whether their neurodevelopmental outcomes differed based on exposure to HUM diet or to BOV diet.

## Methods

Six NICUs participated in this retrospective multi-center cohort study to evaluate diet exposure and neurodevelopmental outcomes at 18–22 months CA of former preterm infants with a BW ≤ 1250 g from 2006 to 2010. Each site’s Institutional Review Board approved this study. Former preterm infants who underwent a routine neurodevelopment assessment at 18–22 CA were identified and included in this study. The infants were then retrospectively classified into two cohorts based on the diet they received in the NICU. The HUM cohort received an exclusive human milk-based diet comprised of mother’s own milk, supplemented with donor human milk when mother’s own milk was unavailable, and fortified with donor human milk-based fortifier. The BOV cohort received mother’s own milk fortified with powdered bovine milk-based fortifier or preterm formula if mother’s own milk was not available. Some infants (*n* = 75, 30%) in this cohort study participated in two randomized controlled trials of HUM vs. BOV feeding conducted during the NICU hospitalization. [1, 2] The remaining study infants were identified if they had a BSID-III exam at 18–22 months CA and their diet information was retrospectively collected for purposes of this study.

Infants in the HUM diet group received fortification with donor human milk-based fortifier at varying enteral feeding thresholds based on institutional feeding protocols, ranging from 60–100 mL/kg/day at initiation, with advancement of feeds by 20 mL/kg/day. Infants in the BOV group received fortification when enteral feeds reached 100 mL/kg/day and were tolerated for one day. All study infants were fed according to standardized nutrition protocols at each institution under the direction of the attending neonatologist. These diets continued until 34–36 weeks postmenstrual age, after which time all infants were transitioned to receive BOV diet, either bovine milk-based fortifier or preterm infant formula depending on availability of mother’s own milk or donor human milk. Infants were discharged on a mixture of diets depending on mother’s own milk supply supplemented with 22 kcal/oz post-discharge infant formula.

Infants were evaluated at 18 to 22 months CA and neurodevelopment was assessed by clinicians as part of routine care at each site using the BSID, Third Edition (BSID-III). The BSID-III, published in 2006, is an individually administered tool designed to assess the developmental functioning of infants, toddlers, and young children aged between 1 and 42 months, and evaluates cognitive, language, and motor skills [15]. Exposure of a HUM or BOV was evaluated retrospectively after neurodevelopmental assessment was completed.

Data retrospectively collected included BW, sex, gestational age, maternal age, incidence of NEC, and anthropometrics at 2 years of age. A medical NEC diagnosis was designated as Bell’s stage IIA or greater [16] with the presence of pneumatosis on abdominal radiograph as determined by a pediatric radiologist. Surgical NEC was defined as requiring surgical intervention within the acute phase of illness. Mother’s own milk intake, donor human milk intake, maternal education, socioeconomic status, and discharge anthropometrics were not obtained.

The chi-squared test for homogeneity was used for all categorical data. Wilcoxon rank-sum tests were used for quantitative data because of the skewness of some of the measurements. A multiple linear regression model was used to adjust for BW, sex, study site and NEC as key covariates that could influence cognitive, language, and motor BSID-III scores. With a minimum of 100 per group and with 90% power and 5% significance, it was possible to detect a group difference for any BSID-III category of 0.46 standard deviations.

## Results

Data from 252 infants with neurodevelopmental assessment who were born in 6 NICUs from 2006 to 2010 were collected (101 infants in the HUM group and 151 infants in the BOV group). Some infants (*n* = 75, 30%) in this cohort study participated in a randomized controlled trial of HUM vs. BOV feeding conducted during the NICU hospitalization [1, 2]. Baseline characteristics are shown in Table 1. There were no statistical differences in gestational age, BW, or sex between the groups. Clinical outcomes from the NICU are shown in Table 1. There was no difference in surgical NEC between groups (*p* = 0.5); however, the HUM group had a lower incidence of medical NEC than the BOV group (2% vs 9%, *p* = 0.03, respectively). At follow-up, there were no differences in weight, length, or head circumference between the HUM group and the BOV group at 18 to 22 months CA (Table 2).

**Table 1:** Baseline demographics and NICU outcomes.

	HUM (*n* = 101)	BOV (*n* = 151)	*p* value
Gestational Age (weeks)^a^	27.5 ± 2.4	27.8 ± 2.4	0.37
Birth Weight (g)^a^	908 ± 127	910 ± 209	0.92
Male (%)	56	46	0.13
Mother’s Age (years)^a^	30.5 ± 6.9	28.8 ± 6.6	0.08
Necrotizing Enterocolitis, Medical (%)	2	9	0.03
Necrotizing Enterocolitis, Surgical (%)	0	1.4	0.51

^a^Mean ± SD.

**Table 2:** Outcomes at 18–22 months of Age.

	HUM (*n* = 101)	BOV (*n* = 151)	Unadjusted *p* value	Adjusted *p* value
Weight (kg)^a^	10.6 ± 1.4	10.7 ± 1.7	0.69	0.64
Length (cm)^a^	82.4 ± 4.5	83.2 ± 4.1	0.38	0.66
Head Circumference (cm)^a^	47.0 ± 2.0	47.1 ± 1.8	0.75	0.60
BSID-III Cognitive Composite*	96.5 ± 15.1	89.6 ± 14.1	0.001	0.0001
BSID-III Language Composite*	85.5 ± 15.0	82.2 ± 14.1	0.15	0.09
BSID-III Motor Composite*	92.9 ± 11.7	91.4 ± 14.6	0.45	0.32

*Mean ± SD, *p* value adjusted for birth weight, sex, center and necrotizing enterocolitis.

^a^Mean ± SD.

All BSID-III scores were adjusted for BW, sex, site and NEC. Mean BSID-III cognitive scores were significantly higher in the HUM group compared to BOV group (96.5 ± 15.1 vs 89.6 ± 14.1, adjusted *p* = 0.001, Table 2). There was an estimated difference of approximately seven points. Mean BSID-III language scores were 85.5 ± 15.0 in the HUM group compared to 82.2 ± 14.1 in the BOV group (adjusted *p* = 0.07). Mean BSID-III motor scores were 92.9 ± 11.7 in the HUM group compared to 91.4 ± 14.6 in the BOV group (adjusted *p* = 0.18).

## Discussion

There are limited post-discharge neurodevelopmental outcome data for infants who were fed a HUM diet in the NICU [4–6]. In this retrospective multi-center cohort study in infants ≤ 1250 g BW who underwent neurodevelopmental assessment at 18–22 months CA, we compared neurodevelopmental outcomes based on exposure to a HUM or BOV diet. We found that extremely preterm infants who had neurodevelopmental assessment and were fed a HUM diet had higher cognitive BSID-III scores and lower incidence of medical NEC compared to infants fed a BOV diet.

Ehrenkranz el al determined that higher weight gain velocity for preterm infants while in the NICU was more protective against cerebral palsy (CP), BSID scores <70, and neurodevelopmental impairment [17]. A 2016 systematic review concluded that early enteral nutrition may reduce neurodevelopmental impairment among very low birth weight (VLBW) infants although studies were heterogeneous and nutritional interventions differed between studies [18]. In our study, although infants received different diets in the NICU, infants had similar weight, length and head circumferences at follow-up. While growth was similar, infants fed HUM diet had higher BSID-III cognitive scores.

A recent Canadian study of 109 VLBW infants did not find any differences in BSID-III scores between infants fed a donor human milk-based fortifier or bovine milk-based fortifier [4]. While this study does provide randomized prospective data, the use of mother’s own milk was high in both study groups leading to less exposure to bovine milk-based products and included infants 1250–1500 g BW who were not as high a risk of poor neurodevelopmental outcomes. Many NICUs do not have such high rates of mother’s own milk use including one of the sites in this study [6]. Infants in the Canadian study also had feedings advanced more gradually with later fortification of enteral nutrition around day of life 16, compared to other published exclusive human milk-based diet cohorts [1, 19]. These differences limit the applicability of the study to a diverse preterm infant population and current feeding strategies in many NICUs.

Colacci et al. also evaluated growth and development of preterm infants before and after the introduction of a HUM diet in their NICU [5]. Cognitive scores from BSID-III testing were similar between infants who received the BOV diet including both bovine milk-based fortifier and infant formula and those who received the HUM diet [5]. However, the HUM diet fortifier was started later compared to our study sites, which may explain some of the difference in findings. As found in our study, Colacci et al. found similar growth between groups.

Recently, Rahman et al. evaluated neurodevelopmental outcomes in premature infants fed a HUM diet. Fortification of +4 kcal/oz began when feeds reached 80 mL/kg/day and were advanced at the attending physician’s discretion [20]. Reported BSID-III scores appear similar to our cohort for cognitive (93 vs 96.5), language (89 vs 85.5), and motor scores (91 vs 92.9), respectively. Rahman et al. did not find any difference between BSID-III scores of babies with extrauterine growth restriction, defined as weight <10th percentile at NICU discharge, and those without extrauterine growth restriction, suggesting that weight gain velocity may not relate to neurodevelopment in infants fed a HUM diet [20].

Numerous factors affect a preterm infant’s developmental outcomes. In addition to type of feeding used, treatment options such as surgery or medications may also play a role. One recent systematic review of studies on NEC survivors found that the most common neurodevelopmental impairment associated with NEC was CP (18%) [19]. Furthermore, the review defined neurological impairment as a significant deviation or loss of neurodevelopmental function, resulting in below average performance on neurological, cognitive or developmental assessment tools. The degree of neurodevelopmental impairment correlated to the severity of gut injury, with worse outcomes in infants with surgical NEC versus medical NEC (43% vs. 27%, respectively) [21]. There were fewer cases of medical NEC in the HUM group of our study, not surgical NEC, confirming previously published reports of a benefit of a HUM diet on NEC outcomes [1, 2, 22, 23]. However, in the current cohort, the infants without NEC had higher BSID-III cognitive scores if they received the HUM diet compared to the BOV diet. This demonstrates that a HUM diet may have additional mechanisms involved with health and neurodevelopment aside from NEC outcomes.

Although the BSID is the most commonly and widely used measure of cognitive function in infants ages 18–36 months at high risk [24], there are limitations to applying the BSID-III to preterm infants. Some studies conclude that results < 70 are predictive of later school age functioning; however, evidence is not overwhelming [25, 26]. Our study cohort of 252 preterm infants is large for a neurodevelopmental outcomes study and infants were from six different geographic NICUs. Study infants and exposure of a HUM or BOV were identified and evaluated retrospectively after neurodevelopmental assessment was completed. Our study was intended to determine if there were differences in neurodevelopmental scores. With a minimum of 100 per group and with 90% power and 5% significance, it was possible to detect a group difference for any BSID-III category of 0.46 standard deviations. Our sample size was 252 infants in total, however, the BOV cohort was over represented by 50 study subjects compared with HUM cohort. The groups were uneven because infants were identified for the study based on if they underwent a routine neurodevelopment assessment at 18–22 months CA. For this reason, the number of infants that were lost to follow-up at the neurodevelopment clinic is unknown. This may have contributed to selection bias. In addition, clinicians performing the BSID-III exams as part of clinical care, were not blinded to infants’ diet. Infants were retrospectively grouped based on exposure to a HUM or BOV diet. To our knowledge, there were no major changes to practice that would affect neurodevelopmental outcomes during the study time period, however we many have not been able to account for all of the factors that affect outcomes.

Limitations of our study include a lack of data on the percent intake of diet (mother’s own milk, donor human milk, and preterm formula), days on parenteral nutrition, or days to full enteral feeds. Another limitation is that the BOV group may not reflect feeding practices in in contemporary NICUs that use donor human milk rather than preterm formula early in the neonatal period. However, all study infants were fed according to standardized nutrition protocols at each institution. Due to the retrospective nature of our study, our analyses did not include other important covariates that have been associated with provision of mother’s own milk and neurodevelopment, such as socioeconomic status or maternal education, as these variables were not routinely gathered during the follow-up visit.

## Conclusion

An exclusive human milk-based diet was associated with higher cognitive neurodevelopmental assessment scores in extremely preterm infants who underwent neurodevelopmental assessment at 18–22 months CA.

## Data Availability

The datasets generated during and/or analysed during the current study are available from the corresponding author on reasonable request.
